# Clinical Outcomes of Robotic Stereotactic Accelerated Partial Breast Irradiation: A Structured Literature Review

**DOI:** 10.7759/cureus.111198

**Published:** 2026-06-20

**Authors:** Yuri A Gerasimov, Alina V Mazurok, Konstantin Nikitin, Valentin A Vaskovskii, Natalia Y Germanovich, Anatoliy Y Popov

**Affiliations:** 1 Radiation Oncology, The National Medical Research Center of Surgery named after A. Vishnevsky, Moscow, RUS; 2 Oncology, The National Medical Research Center of Surgery named after A. Vishnevsky, Moscow, RUS; 3 Medical Oncology, The National Medical Research Center of Surgery named after A. Vishnevsky, Moscow, RUS

**Keywords:** breast cancer, breast-conserving surgery, cyberknife, radiosurgery, robotic partial breast irradiation, stereotactic body radiotherapy

## Abstract

Accelerated partial breast irradiation (APBI) has emerged as an evidence-based alternative to whole-breast irradiation (WBI) in appropriately selected patients with early-stage breast cancer. Within this landscape, robotic stereotactic APBI delivered with the CyberKnife (CK) system (Accuray Incorporated, Madison, WI) represents a technologically refined external-beam implementation, integrating non-isocentric beam arrangements, high-frequency image guidance, and optional fiducial-based respiratory tracking that allow steep dose gradients and reduced geometric margins. This structured literature review synthesizes the available clinical, dosimetric, and technical evidence on CK-APBI to define its current role among contemporary APBI modalities.

A structured review of peer-reviewed studies published between 2011 and 2025 reporting clinical or dosimetric outcomes of CK-based APBI in early-stage breast cancer was performed. Eligible studies included pilot feasibility reports, phase I/II prospective trials, retrospective cohorts, comparative analyses, and dosimetric evaluations. Endpoints of interest were ipsilateral breast tumor recurrence (IBTR), acute and late toxicity, cosmetic outcomes, and dose to organs at risk (heart, lungs, uninvolved breast). Patient selection, treatment technique (fiducial placement, simulation, target delineation, motion management, dose-fractionation), and CyberKnife platform evolution (Iris, InCise multileaf collimator) were also evaluated.

Across cohorts treated predominantly with 30 Gy in five consecutive daily fractions, CK-APBI achieved low IBTR rates (~0-2%) at intermediate and five-year follow-up, with predominantly grade ≤1 toxicity and excellent or good cosmesis in 80-96% of patients. Phase I dose escalation suggested that late grade 3 fat necrosis correlated with larger planning target volume, reinforcing the importance of strict volume control. Recent data, including a 74-patient cohort using non-invasive skin-fiducial tracking and a long-term cohort with five-year follow-up, confirmed durable local control and preserved cosmesis. Dosimetric analyses demonstrated favorable sparing of uninvolved breast tissue (V50 ~18-25%, V100 <10%), low ipsilateral lung dose, and reduced cardiac exposure in left-sided tumors compared with interstitial brachytherapy. Multileaf collimator-based delivery improved efficiency relative to Iris collimation. Fiducial-based image guidance and respiratory tracking enabled the clinical target volume to planning target volume margin reduction to ~2-3 mm, translating into smaller treated volumes and a plausible mechanistic basis for the favorable toxicity and cosmetic profiles observed.

CK-APBI is a clinically mature and technically refined APBI option for appropriately selected early-stage breast cancer patients, with oncologic and cosmetic outcomes broadly concordant with randomized APBI evidence. Margin reduction enabled by image guidance and respiratory tracking provides a distinct technical advantage in limiting intermediate-dose exposure to uninvolved breast tissue and critical organs. However, the evidence base remains predominantly single-institution and non-randomized, with heterogeneous contouring and reporting and limited follow-up beyond five years. Prospective multicenter studies with standardized endpoints are needed to define the relative role of robotic stereotactic APBI among contemporary partial-breast options.

## Introduction and background

Breast-conserving surgery (BCS), followed by adjuvant radiotherapy, is an established standard for early-stage breast cancer, providing equivalent overall survival to mastectomy while preserving breast anatomy. Whole-breast irradiation (WBI) reduces ipsilateral breast tumor recurrence (IBTR) and improves long-term local control, historically delivered with conventional fractionation over five to six weeks and increasingly with moderate hypofractionation over approximately three weeks.

Despite the efficacy of WBI, patterns of failure consistently indicate that most in-breast recurrences arise within the index quadrant, commonly adjacent to the original tumor bed. This observation, reinforced by long-term randomized trial follow-up and clinicopathologic mapping, provides the rationale for partial breast irradiation (PBI), which restricts treatment to the region at highest risk of residual microscopic disease [1-4]. Accelerated partial breast irradiation (APBI) builds on this concept by shortening overall treatment time and potentially reducing dose to uninvolved breast tissue and adjacent organs.

Randomized trials have established APBI as an evidence-based alternative to WBI in appropriately selected low-risk patients. The Florence trial demonstrated long-term local control with IMRT-based APBI (30 Gy in five daily fractions) comparable to WBI, while reducing toxicity and improving cosmesis [1]. The RAPID trial confirmed non-inferiority of 3D conformal radiation therapy (3D-CRT) APBI relative to WBI for IBTR, though cosmetic outcomes appeared sensitive to technique and fractionation [2]. The IMPORT LOW trial further supported partial-breast approaches in carefully selected populations [3]. Consequently, APBI is now integrated into major international guidelines, including the American Society for Radiation Oncology (ASTRO), European Society for Radiotherapy and Oncology (ESTRO)-Advisory Committee on Radiation Oncology Practice (ACROP), and National Comprehensive Cancer Network (NCCN) guidelines, emphasizing strict risk-adapted eligibility [5-7].

Within this landscape, robotic stereotactic APBI delivered with the CyberKnife (CK) system (Accuray Incorporated, Madison, WI) represents a technologically refined implementation of external-beam APBI. The platform integrates non-isocentric beam arrangements and high-frequency image guidance, with optional fiducial-based respiratory tracking, allowing steep dose gradients and reduction of geometric margins [8-15]. From a radiobiological standpoint, clinical outcomes from hypofractionation and APBI trials, together with modeling analyses, suggest that the α/β ratio for breast cancer may be relatively low (approximately 3-4 Gy), providing a plausible biological framework for ultra-hypofractionation in selected patients when geometric precision is ensured [1-3,8].

## Review

Literature search and study selection

This review was designed as a structured narrative review rather than a formal systematic review or meta-analysis. A PRISMA-informed approach was used to improve transparency in literature selection [16]. Publications were screened independently by two authors according to predefined eligibility criteria, with disagreements resolved by discussion. Eligible sources included peer-reviewed clinical, dosimetric, technical, and guideline publications addressing APBI and CyberKnife-based delivery. Overall, 23 relevant sources were included in the qualitative synthesis.

Given the substantial heterogeneity of the available evidence, including differences in study design, patient selection, dose fractionation, target delineation, motion management strategy, follow-up duration, and reported endpoints, formal pooled statistical analysis or meta-analysis was not performed. The included studies were predominantly single-institutional and observational; therefore, the findings were synthesized descriptively, with emphasis on clinically relevant ranges of IBTR, toxicity, cosmetic outcomes, and dosimetric parameters rather than pooled effect estimates.

Indications and patient selection

Appropriate patient selection remains fundamental to maintaining oncologic safety with APBI. ASTRO, ESTRO-ACROP, and NCCN recommendations define eligibility based on clinical, pathological, and biological risk factors [5-7]. In general, suitable candidates include women aged ≥40-50 years with unicentric invasive carcinoma typically ≤2-3 cm (pT1-early pT2), negative margins (≥2 mm), node-negative disease (pN0), and no extensive lymphovascular invasion. Favorable biology (e.g., hormone receptor (HR)-positive and HER2-negative) and non-multicentric disease further support eligibility [5-7].

Caution is recommended for younger age (40-49 years), tumors >2-3 cm, high-grade lesions, estrogen receptor (ER)-negative disease, or marginal resections where local recurrence risk may be higher. Patients with positive margins, nodal involvement, multicentric disease, BRCA1/2 mutation, prior ipsilateral breast irradiation, or HER2-positive tumors without adequate systemic therapy are generally considered unsuitable outside trials [5-7]. These selection criteria are consistent with the populations included in randomized APBI trials demonstrating non-inferior local control relative to WBI [1-3]. Accordingly, outcomes of robotic stereotactic APBI should be interpreted within the framework of guideline-based selection.

Treatment technique (CyberKnife APBI)

Fiducial Placement and Target Localization

CK-APBI is typically delivered four to 12 weeks after lumpectomy following confirmation of pathological eligibility and wound healing [5-7]. Accurate cavity localization is essential for stereotactic delivery and for managing respiratory motion when tracking is applied [15]. Surgical clips placed at lumpectomy assist in delineating the postoperative cavity, particularly when the seroma is minimal.

For intrafraction motion management, many CK-APBI protocols use three to five implanted gold fiducial markers positioned in proximity to the cavity to enable real-time tracking [8-11,15,17]. Postoperative ultrasound-guided implantation is commonly preferred after final pathology confirmation. Recommended geometric principles include placement within breast parenchyma ≥1-2 cm from the cavity margin, inter-marker separation ≥18-20 mm, overall spatial spread typically <10 cm, and non-coplanar distribution to reduce overlap on orthogonal imaging and minimize rotational uncertainty during tracking [15]. Post-implant mammography or CT is performed to verify three-dimensional positioning prior to simulation and planning [8,11,17].

Across key clinical experiences, tracking feasibility is supported by the use of ultrasound-guided or CT-guided fiducials (Rahimi et al., Obayomi-Davies et al., Vermeulen et al., and later cohorts similarly) [8-12,17]. Stable fiducial geometry and robust tracking enable reduced geometric margins in tracking-enabled workflows [10-12,15,17].

In addition to implanted radiopaque fiducials, a non-invasive respiratory tracking strategy has recently been reported using skin fiducial markers coupled with internal surgical clips as surrogates for the lumpectomy cavity position. In a retrospective cohort of 74 patients treated with CK-APBI (30 Gy in five consecutive daily fractions), Wei et al. used five to six skin markers placed within 5 cm of the tumor bed and validated their motion correlation with internal surgical clips on respiratory-phase imaging, reporting correlation coefficients close to 1 for multi-axis displacement [18]. This approach is of particular interest in patient populations where invasive implantation may be less acceptable or technically challenging (e.g., smaller breast volumes), and it provides a pragmatic alternative for motion management while retaining the advantages of CyberKnife image guidance.

Simulation, Immobilization, and Image Guidance

Patients are simulated supine with ipsilateral arm elevation, using immobilization devices to promote reproducibility. Thin-slice CT imaging is typically acquired for high-resolution cavity delineation and stereotactic planning. When cavity definition is uncertain, MRI may be co-registered to improve visualization (e.g., in minimal-seroma cases) [8,11,17]. The CyberKnife system relies on frequent orthogonal imaging during delivery; in tracking-enabled cases, internal fiducial position is correlated with respiratory motion to compensate for intrafraction displacement [15].

Target Delineation (CTV/PTV)

In postoperative APBI, a gross tumor volume is not defined; target volumes include clinical target volume (CTV) and planning target volume (PTV). The cavity is delineated using clips, seroma, and preoperative imaging correlation; MRI can assist when CT visualization is limited [8,11,17]. Most CK series define the CTV as the cavity expanded by ~10-15 mm to cover potential microscopic extension, cropped near the skin surface and limited posteriorly at the pectoral fascia/chest wall interface [5-6,8,10-12,17]. These principles align with APBI guidance and anatomical constraints intended to preserve cosmesis [5,6].

PTV margins depend on motion management. With fiducial-based respiratory tracking, PTV expansions of ~2-3 mm are feasible; earlier approaches or reduced tracking confidence may use up to 5 mm [8,10-12,15,17]. The capacity to minimize PTV margins is a key differentiator of stereotactic APBI compared with conventional external beam APBI approaches that require larger motion/setup expansions [15,19].

Dose and Fractionation

The dominant prescription across the CK-APBI series is 30 Gy in five fractions delivered on consecutive days [8-14,20]. Alternative schedules include 25 Gy in four fractions in phase II experience [17]. Phase I escalation explored 30-40 Gy in five fractions; rare late grade 3 toxicity was associated with larger PTV volumes, suggesting that both dose and treated volume influence late effects in ultra-hypofractionated delivery [10]. These observations support the practical convergence toward 30 Gy/5 as a biologically effective yet clinically tolerable schedule.

Clinical outcomes

Early Clinical Experience (2011-2018)

Table 1 summarizes the key clinical studies of CyberKnife-based stereotactic APBI, including study design, sample size, dose/fractionation, follow-up, and main oncologic and toxicity outcomes.

**Table 1:** Clinical studies of CyberKnife‑based APBI (2011-2025). APBI: accelerated partial breast irradiation; IBTR: ipsilateral breast tumor recurrence; WBI: whole-breast irradiation; PTV: planning target volume.

Study	Year	Study design	n	Dose/fractionation	Follow-up	Main oncologic outcomes	Toxicity/cosmesis
Vermeulen et al. [8]	2014	Pilot feasibility study	9	30 Gy/5 fx or 34 Gy/10 fx	Short	No local recurrence reported	Mostly grade 1 toxicity, excellent/good cosmesis
Obayomi-Davies et al. [9]	2016	Early clinical series	10	30 Gy/5 fx	1.3 years	No IBTR	Acute toxicity ≤ grade 2
Rahimi et al. [10]	2017	Phase I dose‑escalation	75	30-40 Gy/5 fx	Median of 13.5-49.9 months across 5 cohorts	Feasible stereotactic APBI	Rare late grade 3 fat necrosis; associated with larger PTV
Lozza et al. [11]	2018	Prospective pilot	20	30 Gy/5 fx	~2 years	No IBTR	Grade ≤2 toxicity, excellent/good cosmesis
Mészáros et al. [17]	2020	Phase II prospective	27	25 Gy/4 fx	Early	100% local control	Minimal acute toxicity
De la Pinta & Fernández-Lizarbe [21]	2022	Literature review	28 studies	Various (mainly 30 Gy/5 fx)	–	High local control across stereotactic APBI literature	Favorable cosmesis ≥80–90%
Jaysing et al. [12]	2023	Retrospective cohort	50	30 Gy/5 fx	4.7 years	1 IBTR (~2%)	96% excellent/good cosmesis
Byun et al. [13]	2023	Prospective comparative cohort	103 APBI	30 Gy/5 fx	Intermediate	Comparable tumor control vs. WBI	Lower dermatitis and fibrosis vs. WBI
Wei et al. [18]	2025	Retrospective cohort	74	30 Gy/5 fx	26.5 months	IBTR 2.7%	No ≥ grade 2 toxicity; dermatitis most common acute event
Sherman [20]	2025	Long‑term cohort	54	30 Gy/5 fx	~5 years	IBTR ~2%	Preserved long‑term cosmesis

Early feasibility and pilot studies established CK-APBI as deliverable with favorable early toxicity and cosmesis. Vermeulen and Haas reported early outcomes with 30 Gy/5 or 34 Gy/10, observing no IBTR at early follow-up, predominantly grade 1 toxicity, and high rates of excellent/good cosmetic outcomes [8]. Obayomi-Davies et al. reported no local recurrences with minimal acute toxicity and stable cosmesis through follow-up extending to approximately 1.3 years [9]. Rahimi et al. demonstrated the feasibility of 30-40 Gy/5; rare late grade 3 toxicity (fat necrosis) occurred predominantly in patients with larger PTV volumes (≥124 cm³), highlighting a potential volume-effect relationship [10]. Lozza et al. treated 20 patients with 30 Gy/5 and reported no IBTR and only grade ≤2 toxicity with excellent/good cosmesis in all patients [11]. Collectively, early reports supported feasibility, acceptable safety, and promising local control in selected populations [8-11].

Contemporary Evidence (2020-2025)

More recent cohorts strengthen the evidence base by increasing sample size and follow-up duration. Mészáros et al. reported phase II outcomes with 25 Gy/4, demonstrating 100% local control at analysis, minimal acute toxicity, and no late grade ≥2 events [17]. De la Pinta and Fernández-Lizarbe summarized 28 stereotactic APBI series, reporting consistently high local control, predominantly grade 1 acute toxicity, and favorable cosmesis (≥80-90%), although evidence was largely observational and heterogeneous [21].

Jaysing et al. provided intermediate-term outcomes for 50 patients treated with 30 Gy/5, reporting one local recurrence (~2%) at a median of 4.7 years and a single late grade 3 event, with excellent/good cosmesis in 96% [12]. Byun et al. reported a prospective comparative cohort showing lower dermatitis rates and reduced fibrosis in uninvolved quadrants compared with WBI, supporting the clinical relevance of limiting the dose to non-target breast tissue [13]. Wei et al. reported comparable clinical control between CK-APBI and interstitial brachytherapy with significantly lower mean heart dose and reduced low-dose cardiac volumes for CK delivery, suggesting a potential advantage in left-sided or cardiac-proximate anatomy [14]. The longest follow-up presented to date was by Sherman (ASTRO 2025), reporting ~2% local recurrence with preserved long-term cosmesis at five years in 54 patients treated with 30 Gy/5 [20].

Further contemporary evidence includes a large single-institution Asian cohort employing a non-invasive tracking strategy. Wei et al. retrospectively evaluated 74 patients treated with CK-APBI to 30 Gy in five consecutive daily fractions using skin fiducial markers for respiratory tracking, with a median follow-up of 26.5 months [18]. Ipsilateral breast tumor recurrence occurred in two patients (2.7%), with no reported grade ≥2 toxicity and no cardiopulmonary events. Grade 1 radiation dermatitis was the most common acute event (48.6%), whereas grade 1 breast fibrosis represented the most frequent late toxicity (12.2%).

Dosimetric Analysis and Clinical Correlation

CyberKnife planning achieves PTV coverage consistent with stereotactic APBI requirements. Xu et al. reported a mean PTV coverage of 95.7% across target volumes 39-241 cm³, supporting the ability of CK delivery to maintain conformality across a wide range of cavity sizes [19]. Lozza et al. also documented acceptable minimum target dose relative to prescription, reinforcing adequate coverage and dose gradients in clinical implementation [11].

Dose to uninvolved breast tissue is clinically relevant because intermediate-dose exposure correlates with fibrosis and cosmetic outcomes. In published CK planning data, V50 and V100 values were relatively low: Xu et al. reported V50 ~23% and V100 ~9%, while Lozza et al. reported even lower values (V50 18.6%, V100 0.6%) [11,19]. These indices provide a plausible mechanistic link to the favorable cosmesis reported in clinical series, particularly when margin reduction reduces treated volume.

Cardiac sparing is a key concern in left-sided breast cancers. Wei et al. demonstrated substantially lower mean heart dose with CK-APBI compared with interstitial brachytherapy (107 vs. 204 cGy) and reduced heart V_150cGy_ (24% vs. 60%), suggesting that robotic delivery can reduce cardiac exposure in selected anatomical scenarios [14]. Xu et al. reported heart V5 values up to 9.4% in left-sided cases, consistent with a limited low-dose “tail” and underscoring the importance of meticulous plan optimization for cardiac structures [19].

Pulmonary dose is typically low with CK-APBI. Xu et al. reported very low ipsilateral lung V30 (~1.3%) [19], supporting the expectation of low pneumonitis risk in modern APBI practice. Comparative dosimetric analyses have shown that CK-APBI can achieve favorable organs at risk (OAR) sparing relative to multicatheter brachytherapy in selected geometries (notably heart dose), while brachytherapy may reduce peak dose to superficial structures (skin/ribs) in certain anatomies, highlighting technique selection based on cavity location and organ proximity [14,22].

Finally, motion management influences treated volume. Tracking-enabled delivery allows smaller PTV margins, reducing total irradiated breast volume. Mészáros et al. reported reduced PTV volumes with tracking relative to non-tracking approaches, supporting the concept that motion compensation contributes directly to improved dosimetry and potentially cosmetic endpoints [17]. Collectively, dosimetric findings provide a mechanistic explanation for the favorable toxicity and cosmetic profiles observed across clinical cohorts.

Dosimetric outcomes in CK-APBI should also be interpreted in the context of evolving CyberKnife hardware platforms. Earlier studies were primarily performed on CyberKnife VSI systems using fixed circular collimators or the Iris™ variable aperture collimator, whereas newer treatments are increasingly delivered on CyberKnife M6 platforms equipped with the InCise™ multileaf collimator (MLC). Goggin et al. demonstrated that both CyberKnife Iris and MLC-based APBI plans achieved superior dose conformity relative to 3D-CRT, with reduced high-dose exposure to surrounding uninvolved breast tissue. Mean ipsilateral breast V50% values were substantially lower with CyberKnife techniques (approximately 24-25%) compared with 3D-CRT (56.2%), supporting improved normal breast sparing. Although 3D-CRT produced lower lung low-dose exposure (V5%), CyberKnife plans incorporated real-time motion tracking, potentially allowing greater geometric precision and margin reduction. Importantly, MLC-based delivery reduced monitor units and shortened treatment time by approximately 50% relative to Iris-based plans, supporting improved delivery efficiency and potentially reduced intrafraction motion risk [23].

Discussion

The available evidence suggests that CK-APBI achieves oncologic outcomes broadly consistent with established APBI approaches, while offering a distinct technical pathway to reduce treated volume. Reported IBTR rates across CK cohorts (~0-2% at intermediate follow-up) align with outcomes from randomized APBI trials in selected low-risk populations [1-3], though it must be emphasized that CK data remain largely observational. The convergence of clinical outcomes with randomized APBI evidence supports the interpretation that robotic stereotactic delivery represents a refinement of the APBI paradigm rather than a deviation from its evidence base.

A key differentiator is margin philosophy. In conventional external-beam APBI (e.g., 3D-CRT as used in the RAPID trial [2]), geometric expansions are generally larger to compensate for setup uncertainty and respiratory motion, which may increase intermediate-dose exposure to uninvolved breast tissue and adversely affect cosmesis in some settings [2,24]. In contrast, CK-APBI leverages frequent image guidance and, when enabled, fiducial-based respiratory tracking to reduce PTV margins and limit intermediate-dose spread, an effect reflected in low V50/V100 values and favorable clinical cosmesis in multiple cohorts [11-13,18].

Radiobiologically, ultra-hypofractionated schedules such as 30 Gy in five fractions are supported by clinical outcomes from APBI and hypofractionation trials and by modeling suggesting a relatively low α/β ratio for breast cancer (~3-4 Gy) [1-3,8]. Importantly, phase I escalation indicates that toxicity may increase with treated volume at higher dose levels, reinforcing the clinical importance of careful selection, robust tracking, and strict volume control [10].

From a comparative perspective, CK-APBI offers a non-invasive alternative to brachytherapy and may reduce cardiac exposure in selected patients, particularly with left-sided tumors [14,22]. However, brachytherapy may provide advantages for superficial structure sparing in certain anatomies, and conventional linac-based APBI remains more widely accessible with shorter delivery times. Therefore, technique selection should remain individualized based on anatomy, institutional expertise, and resource availability.

Recent data also broaden the motion-management spectrum beyond implanted fiducials. The non-invasive skin fiducial approach with clip correlation reported by Wei et al. may improve acceptability and feasibility in patients with smaller breast volumes, while maintaining favorable conformity and low cardiopulmonary dose [18]. Whether this strategy can reproducibly match fiducial-based tracking across institutions and longer follow-up remains to be established.

Limitations of the evidence base include modest sample sizes, heterogeneity in contouring and reporting, variable follow-up, and lack of randomized trials directly comparing CK-APBI to other APBI modalities or contemporary WBI schedules. Long-term endpoints beyond five years, particularly late cosmesis, fibrosis, and fat necrosis, require further prospective evaluation. Standardization of target definition, dosimetric reporting, and cosmetic assessment tools would improve cross-study comparability and accelerate broader evidence synthesis.

## Conclusions

Robotic stereotactic APBI delivered with CyberKnife is a clinically mature and technically refined APBI implementation for appropriately selected early-stage breast cancer patients. Across cohorts treated predominantly with 30 Gy in five fractions, reported IBTR rates remain low (~0-2%), with favorable toxicity and durable cosmetic outcomes at intermediate and five-year follow-up. These outcomes are broadly concordant with randomized APBI evidence demonstrating non-inferior local control compared with WBI in low-risk populations.

The biological rationale for ultra-hypofractionation is supported by clinical trial outcomes and modeling suggesting a relatively low α/β ratio for breast cancer (~3-4 Gy), supporting larger fraction sizes when geometric precision is ensured. Fiducial-based image guidance and respiratory tracking enable reduction of CTV-to-PTV margins (~2-3 mm in tracking-enabled workflows), translating into smaller treated volumes and favorable dosimetric profiles for uninvolved breast tissue and critical organs, including the heart and lungs. However, the current evidence base remains predominantly single-institution and non-randomized, with heterogeneity in contouring and reporting and limited long-term follow-up beyond five years. Randomized comparisons specific to robotic stereotactic delivery are lacking. Further prospective multicenter evaluation with standardized reporting and longer follow-up is warranted to define the relative role of CK-APBI among contemporary APBI options.
